# Evaluation of peanut skin and grape seed extracts to inhibit growth of foodborne pathogens

**DOI:** 10.1002/fsn3.503

**Published:** 2017-08-31

**Authors:** Jason Levy, Renee R. Boyer, Andrew P. Neilson, Sean F. O'Keefe, Hyun Sik S. Chu, Robert C. Williams, Melanie R. Dorenkott, Katheryn M. Goodrich

**Affiliations:** ^1^ Department of Food Science and Technology Virginia Polytechnic Institute and State University Blacksburg VA USA

**Keywords:** antimicrobials, foodborne pathogens, grape, peanut, procyanidin

## Abstract

Peanut skin extract (PSE) and grape seed extract (GSE) are derived from waste products in the wine and peanut industries, respectively. Both have high concentrations of polyphenols, known to possess antioxidant and antimicrobial properties. PSE primarily contains “A‐type” procyanidins, while GSE primarily contains “B‐type” procyanidins. These differ structurally, but are both isomers of epicatechin dimers. The objective of this study was to evaluate the antimicrobial effects of PSE containing A‐type procyanidins and GSE containing B‐type procyanidins against select foodborne pathogens (*Listeria monocytogenes*,* Escherichia coli* O157:H7, and *Salmonella* Typhimurium). The minimum inhibitory concentration (MIC) of the two extracts on *L. monocytogenes*,* E. coli* O157:H7, and *S. *Typhimurium was determined using the pour plate method. GSE had a significantly lower MIC (*p* ≤ .05) than PSE for *L. monocytogenes* (GSE = 60.6 ppm, PSE > 68.2 ppm) and *S. *Typhimurium (GSE = 45.7 ppm, PSE = 60.6 ppm), but no difference in inhibition of *E. coli* O157:H7. Since GSE contributed to greater inhibition, GSE extract was fractionated into monomer‐rich (consisting primarily of catechins, epicatechins, and epicatechin gallates) and oligomer‐rich (consisting of dimers, trimers, tetramers, up to decamers) components. Growth curves of all three pathogens in the presence of full extract, monomer and oligomer fractions were compared separately. None of the extracts inhibited *S. *Typhimurium growth. Generally, the extract containing greater oligomer components inhibited growth of *L. monocytogenes* and *E. coli* O157:H7 when compared to the control. Results indicate that an extract with type B procyanidins higher in oligomers may have greater antimicrobial properties.

## 1 INTRODUCTION

In recent years, the demand for the use of natural ingredients has placed an emphasis on identifying natural food preservatives. Traditionally, foods are thermally processed and/or preserved with compounds such as sodium benzoate, nitrites, sodium meta bisulfate, and others that are generally recognized as safe (GRAS) to prevent spoilage organisms and pathogens from growing. The push for more naturally derived compounds for use in food commodities has come from consumers (Aoki, Shen, & Saijo, 2010). The perception that these are “healthier” and “safer” food ingredients has led to the exploration and discovery of antimicrobial compounds from natural sources.

Two materials that have received attention in recent years are grape seed extract (GSE) and peanut skin extract (PSE). One of the benefits of examining these two products is that they are derived from waste products by the industries that produce grape juice/wine and shelled peanuts, respectively (Shi, Yu, Pohorly, & Kakuda, 2003; Yu, Ahmedna, & Goktepe, 2005, 2010). Identifying value in these products could result in less waste, complete utilization of raw products, and provide an all‐natural alternative to synthetic preservatives. These extracts are high in polyphenols, which are a major contributor to antioxidant and antimicrobial properties (Daglia, 2012).

Grape (*Vitis* spp.) seeds contain several active components including catechin monomers ([−]‐epicatechin, [+]‐catechin, etc.), anthocyanins, and procyanidins (Nassiri‐Asl & Hosseinzadeh, 2009). Procyanidins, commonly referred to as proanthocyanidins, are linear oligomers and polymers of catechin monomer residues. Grapes contain “B‐type” procyanidins, in which the monomer residues are linked to each other by a single covalent carbon–carbon bond (Lee & Jaworski, 1987). The antimicrobial properties of GSE have been preliminarily examined. GSE is more effective at reducing growth of Gram‐positive bacteria than Gram‐negative bacteria (Jayaprakasha, Selvi, & Sakariah, 2003). Concentrations of GSE (1%) coinoculated with nisin is effective reducing concentrations of *Listeria monocytogenes* by 9 logs over 3 hr in phosphate‐buffered solution and over 6 logs over 15 hr in tryptic soy broth supplemented with yeast extract (Sivarooban, Hettiarachchy, & Johnson, 2007; Theivendran, Hettiarachchy, & Johnson, 2006).

Peanut skins also contain catechin monomers and procyanidins. However, peanut skins primarily contain “A‐type” procyanidins, in which the monomer residues are linked by two covalent bonds: a carbon–carbon bond and a carbon–oxygen–carbon ether bond (Lou et al., 1999). Peanut skins are traditionally consumed as part of the whole peanut in many areas of the world without adverse effects and would qualify as a GRAS product (Yu et al., 2010). Nuts are a source of antioxidants, polyphenols, and other phytochemicals such as phytosterols and carotenoids (Chen & Blumberg, 2008; King, Blumberg, Ingwersen, Jenab, & Tucker, 2008; Lou et al., 1999; Yu et al., 2005). There has been limited work performed evaluating the efficacy of PSE on inactivating pathogenic bacteria. Yu et al. (2010) found that 0.4% (or greater) PSE completely inhibited *Bacillus subtilis*,* Escherichia coli*,* Salmonella* Typhimurium, and *Staphylococcus aureus* in growth media.

The objective of this study was to evaluate the antimicrobial effects of PSE containing A‐type procyanidins and GSE containing B‐type procyanidins against select foodborne pathogens (*L. monocytogenes*,* E. coli* O157:H7, and *S. *Typhimurium). The extract with the greatest inhibition against the organisms tested was fractionated into monomers and oligomers, and fractions were evaluated for ability to inhibit growth of the three pathogens. Identifying which component of the procyanidin‐rich polyphenol is most effective may inform researchers on the factor that contributes the most to antimicrobial properties. This may help with the selection of other natural extracts using their compound profiles to determine which ones will have the best potential.

## 2 MATERIALS AND METHODS

### 2.1 Chemicals and reagents

Vitaflavan^®^ GSE was purchased from DRT Nutraceutics (Dax, France). Vitaflavan is a water‐soluble extract of *Vitis vinifera* (common wine and table grape) seeds, and manufacturer specifications indicate that it contains 24% w/w monomeric procyanidins, 42% w/w dimeric/trimeric procyanidins, and 10% w/w larger procyanidins (tetramers, pentamers, etc.). LC‐MS grade solvents (water, acetonitrile, and formic acid) and ethyl acetate, acetone, and methanol (all American Chemical Society (ACS) grade) were obtained from VWR International (Radnor, PA). Milli‐Q water was prepared using a Millipore Milli‐Q Gradient system. The standards (+)‐catechin hydrate, (−)‐epicatechin, (−)‐epicatechin gallate, (−)‐epigallocatechin, and (−)‐epigallocatechin gallate were obtained from Sigma (St. Louis, MO). PC dimer B_2_ and trimer C_1_ were obtained from ChromaDex (Irvine, CA). PC dimers B_1_, B_5_, B_2_ gallate, trimer T_2_, tetramer A_2_, pentamer, and hexamer were obtained from Planta Analytica (Danbury, CT).

### 2.2 Preparation of extracts

Extracts were prepared fresh prior to each experiment. GSE was a commercially available extract (Vitaflavan^®^, Les Dérivés Résiniques & Terpéniques, Dax, France). PSE was prepared from crude peanut skins donated by Seabrook Ingredients (Edenton, NC). Extraction methodology followed previously published methods (Adamson et al., 1999; Robbins et al., 2012; Ye et al., 2016) with slight modification. Briefly, 50 g peanut skins were shaken in a sieve to remove dust and combined with 750 ml hexane, mixed thoroughly, and sonicated for 10 min. The slurry was allowed to settle, and hexane containing the fat was decanted and discarded. Defatting was repeated twice more, and residual hexane was air evaporated for 24 hr. The defatted skins were combined with 400 ml acetone:water:acetic acid (70:28:2, v/v/v). The mixture was sonicated (10 min) and the supernatant was placed in a filtered stomacher bag (to aid in the separation of the solids and liquids). The supernatant was then transferred to centrifuge tubes. The peanut skins were added to the stomacher bag with 40 ml of the acetone/water/acetic acid mixture and stomached for 2 min. The liquid was transferred to enough 50 ml centrifuge tubes to accommodate all the supernatant, while the peanut skins remained in the stomacher bag. The process of adding of acetone:water:acetic acid, peanut skins, and stomaching was repeated three more times. After the supernatant from all four homogenates was collected, it was centrifuged at 4,000 rpm for 20 min. The supernatant was then transferred to 1,000 ml flasks (approximately 500 ml into each flask). The contents were placed in the Rotovapor (RV 10, IKA, Wilmington, NC) at 37–40°C, 100 rpm with a vacuum of 20 psi for 3–4 hr (until boiling stopped, indicating the removal of the acetone fraction). The remaining liquid (predominately water) was placed into plastic cups and frozen for 12 hr. The frozen extract was then placed into a 1,000 ml beaker. The contents were freeze dried for 3 days on a FreeZone 1 L freeze drier (Labconco, Kansas City, MO).

### 2.3 Preparation of inoculum

Cultures were activated prior to each experiment. *Salmonella* Typhimurium (ATCC 14,028) and *E. coli* O157:H7 (1994 beef outbreak isolate) were stored on TSA (typtic soy agar; Becton, Dickinson and Company, Sparks, MD) slants and *L. monocytogenes* (LCDC) was stored on TSA + 0.6% yeast extract (TSAYE; *L. monocytogenes*) slants. One loopful of each stored culture was transferred to tubes of 10 ml tryptic soy broth (TSB; Becton, Dickinson and Company, Sparks, MD) or TSBYE and incubated at 37°C for 24 hr. Cells from each tube were then centrifuged at 1,192*g* for 10 min at 22°C, the supernatant was decanted, and the pellet was resuspended in 10 ml sterile 0.1% peptone water (SPW). The pellet was washed twice more with SPW to yield a bacterial inoculum of approximately 8 log CFU/ml. This procedure was followed up for each bacterial culture separately. Each inoculum was then serially diluted in SPW to reach a final inoculum concentration of 5 log CFU/ml. Final concentrations of inoculum were confirmed by serially diluting the inoculum in SPW and plating on TSA or TSAYE.

### 2.4 Determining minimum inhibitory concentration of GSE and PSE

The methods used in this study were similar to those described in Jayaprakasha et al. (2003); however, lower concentrations were used. The agar dilution method was used to determine the minimum inhibitory concentration (MIC) of each extract/bacterium combination. Quantities of GSE and PSE were dissolved in sterile deionized (DI) water at concentrations of 0.025–0.15 g/100 ml (250–1,500 ppm). One ml of each concentration of GSE or PSE was separately dispensed onto sterile Petri dishes and 20 ml of molten TSA (40–42°C) was added, resulting in final concentrations of 11.4–68.2 ppm GSE or PSE in the agar. Next, 100 μl of each pathogen solution (5 log cfu/ml, described above) was dispensed onto duplicate sterile Petri dish plates for each concentration of GSE or PSE and swirled for 20 s to mix thoroughly. Plates were incubated at 35°C for 24 hr (*E. coli* O157:H7 and *S. *Typhimurium) or 48 hr (*L. monocytogenes*). A negative control treatment was prepared by pour plating 20 ml of molten TSA with each pathogen separately (no PSE or GSE). A positive control treatment was prepared by the same procedure used for GSE or PSE, but using 1 ml of 0.05 g/100 g (500 ppm) *trans*‐cinnamaldehyde (final concentration 23.8 ppm in the agar) (Mahmoud, 1994). Following incubation, plates were observed for the presence or absence of growth. The MIC is defined as the lowest concentration of the compound which completely inhibited growth of the bacterium on the plate. The experiment was performed in triplicate.

### 2.5 Determination of total phenolics

Peanut skin extract and GSE were diluted to 0.0, 0.1, 0.2, 0.3, 0.4, and 0.5 g/L water. This was done by mixing 5 mg extract with 50 ml water (for PSE, 25 ml of acetone was added). The standard was compared with gallic acid with the same concentrations. Aliquots of 100 μl samples or gallic acid standards were mixed with 2.5 ml 0.2 N Folin–Ciocalteu reagent and vortexed. Then, 2.0 ml saturated sodium carbonate was added and mixed on the vortexer. These solutions were allowed to sit for 2 hr and sample absorbance at 765 nm was measured. Each concentration was done in duplicate.

### 2.6 Fractionation of GSE

Grape seed extract was fractionated according to methods described by Dorenkott et al. (2014) with modification. Briefly, tC18 and C18 Sep‐Pak columns (20 cc, 5 g, Waters, Milford, MA) were connected by Waters adapters to form 2‐stage columns. Columns were preconditioned with by running 10 ml methanol followed by 10 ml of DI water. GSE was dissolved in 70:28:2 acetone:DI water:acetic acid solution (0.2 g/ml) by sonicating for 15 min. GSE solution (1.5 ml) was then loaded onto columns with the use of a vacuum and the nonretained eluent was discarded. Monomeric catechins were eluted using 120 ml diethyl ether and the eluent was set aside. Procyanidins were then eluted using 120 ml methanol and the eluent was set aside. The two eluents containing monomeric catechins and procyanidins were separately dried under rotary vacuum evaporation. The residues were resolubilized in 25 ml water using sonication. The resolubilized fractions were then frozen at −80°C and freeze dried for 2 days, until use in subsequent analysis.

### 2.7 UPLC‐MS/MS analysis

UPLC separations were performed based on our previously reported method (Goodrich & Neilson, 2014) to confirm that the monomer and oligomer fractions were indeed enriched for procyanidin monomers and oligomers, respectively. UPLC was performed on a Waters Acquity H‐class separation module equipped with a Waters Acquity UPLC HSS T3 column (2.1 mm × 100 mm, 1.8 μm particle size). The column temperature was set to 40°C, and the samples were maintained at 10°C. The binary mobile phase system comprised 0.1% (v/v) aqueous formic acid (phase A) and 0.1% (v/v) formic acid in Acetonitrile (ACN) (phase B). Solvents for UPLC0MS/MS were LC‐MS grade (VWR, Radnor, PA). The system flow rate was 0.6 ml/min. Elution was performed based on the following linear gradient: 95% A at 0 min held until 0.5 min, 65% A at 6.5 min, 20% A at 7.5 min held until 8.75 min, 95% A at 8.85 min held until 10.0 min.

MS/MS analysis of column effluent was performed by (−)‐electrospray ionization (ESI) on a Waters Acquity TQD (triple quadruple) mass spectrometer equipped with a Z‐spray electrospray interface. The ESI capillary voltage was −4.25 kV, and the source and desolvation temperatures were 150°C and 400°C, respectively. The desolvation gas and cone gasses were N_2_ at flow rates of 900 L/hr and 75 L/hr, respectively. The MS/MS collision gas was Ar. Data acquisition was carried out with MassLynx software (version 4.1, Waters). MS data collection was set to 10 points/peak with an average peak width of 6 s. The auto‐dwell setting was used to automatically calculate dwell time based on an interscan delay time of 0.02 s for each transition. The Intellistart function of MassLynx was used to develop and optimize multireaction monitoring (MRM) parameters for each compound of interest (Table 1). Compound solutions were directly infused into the ESI source (0.1 mg/ml in MeOH/0.1% formic acid at a flow rate of 50 μl/min) in combination with a background flow of 50% phase A/50% phase B at 0.6 ml min. Intellistart automatically selected the most abundant daughter ion, optimized the source cone voltage and MS/MS collision energy, and generated a single MRM transitions for each compound.

**Table 1:** MS/MS settings for multireaction monitoring (MRM) detection of monomers and procyanidins

Compound	*t*_R_ a (min)	MW (g/mol)	[M − H]^−^ (m/z)^b^	Daughter ion (m/z)	Cone voltage (V)	Collision energy (eV)
PC dimer B_1_	2.68	578.136	577.136	289.105	38	24
(−)‐Epigallocatechin	2.76	306.038	305.038	124.977	40	22
Unknown PC dimer 1^c^	2.92	578.136	577.136	425.102	36	16
(+)‐Catechin	2.99	290.028	289.028	245.057	36	14
PC trimer T_2_	3.09	866.218	865.218	289.102	36	48
Unknown PC dimer 2^c^	3.29	578.136	577.136	425.102	36	16
PC dimer B_2_	3.34	578.136	577.136	425.102	36	16
(−)‐Epicatechin	3.63	290.092	289.092	245.056	42	12
(−)‐Epigallocatechin gallate	3.67	458.038	457.038	168.982	34	16
PC trimer C_1_	3.82	866.218	865.218	287.085	46	32
PC cinnamtannin tetramer A_2_	3.97	1154.808	576.404	125.020	26	34
PC dimer B_2_ gallate	3.99	730.164	729.164	407.129	42	32
Unknown PC dimer 3^c^	4.07	578.136	577.136	425.102	36	16
PC pentamers	4.10	1442.820	720.410	125.022	26	44
PC hexamers	4.23	1731.038	864.519	125.020	32	56
(−)‐Epicatechin gallate	4.60	442.076	441.076	168.968	38	18
PC dimer B_5_	4.64	578.136	577.136	289.107	30	26

a Retention time.

b m/z values represent monoisotopic masses detected by Intellistart; all MRMs used singly charged parent ions except for cinnamtannin tetramer A_2_, pentamers, hexamers, heptamers, octamers, which were doubly charged ([M** − **2H]^2−^), and nonamers and decamers, which were triply charged ([M** − **3H]^3−^).

c Likely procyanidin dimers B_3_, B_4_, and either B_6_, B_7_, or B_8_.

All compound peaks were processed and quantified using the TargetLynx function of MassLynx software. Quantification parameters for native compounds are shown in Table 1. Compounds were quantified based on external standard curves of authentic standards; compounds for which authentic standards were not available were quantified based on external standard curves of similar compounds.

### 2.8 Comparing growth inhibition of GSE fractions

Fractionated GSE was evaluated using methods previously described by Sarnoski, Boyer, and O'Keefe (2012). Briefly, the antimicrobial effect of the fractions was evaluated using a BioScreen Growth Curve machine (Growth Curves USA, Piscataway, NJ, USA). The Bioscreen uses a 100‐well honeycomb plate (Growth Curves USA). Treatments included final concentrations (25, 50, 100, 200 ppm) of each monomer fraction, oligomer fraction, unfractionated GSE. Controls included 100 ppm *trans*‐cinnamaldehyde, and sterile TSB (inoculated and uninoculuated). One plate was used per pathogen to prevent any potential cross contamination. Each plate included five wells per treatment (15 treatments); therefore, for each run 75 wells in the honeycomb plate were filled. Each well was filled with 100 μl of the treatment and 100 μl of the inoculum (previously described) in each well. The plate was shaken, and read every 15 min for 48 hr at 35°C. Three plates per organism were replicated (*n* = 15).

### 2.9 Data and statistical analyses

The growth of *L. monocytogenes*,* E. coli* O157:H7, and *S. *Typhimurium in the varying concentrations of GSE and PSE was performed in duplicate on four separate days. MIC values for each respective bacterium were recorded based on the presence or absence of growth. Error bars were created using standard deviation. For MIC values, *t* tests were performed between GSE and PSE for each organism tested, with correction for multiple comparisons using the Holm‐Sidak method. Significance was defined as *p* < .05.

All BioScreen points were recorded and plotted on Microsoft Office Excel 2007 (Microsoft, Redmond, WA, USA). Microsoft Office Excel 2007 was used to calculate the area under the optical density/time curve (AUC) for each compound. The effect of the treatments on the growth of each organism is shown as a reduction in the AUC when compared to the control. This enabled easy comparison between the different compounds. Significant differences between the AUCs were analyzed with ANOVA and Tukey–Kramer HSD method on JMP Pro 10 (SAS Institute Inc., Cary, NC, USA).

Regression was conducted in Microsoft Excel between absorbance vs. concentrations of standard solutions. The results from the each concentration from each sample were averaged and compared to the standard solution and a percentage was calculated.

## 3 RESULTS

### 3.1 Confirmation of bacterial cultures

Each of the bacterial cultures was confirmed on solid media. Confirmation of *E. coli* O157:H7 on Sorbitol MacConkey agar plates was positive as indicated by growth of colorless colonies. Growth on the XLT‐4 agar plates was positive (black colonies indicating H_2_S production) for the presence of *S. *Typhimurium. Growth on the modified Oxford agar plates was positive (media became black with colony growth) for the presence of *L. monocytogenes*.

### 3.2 Total phenolics count

Grape seed extract contained a significantly greater percentage of total polyphenol contents (96.8 ± 1.04%, w/w, gallic acid equivalents, GAE) compared to peanut skin extract (76.6 ± 7.83%, w/w, GAE).

### 3.3 Minimum inhibitory concentration of GSE and PSE

Throughout the experiment, the negative control plates (inoculated TSA with no extract) had a lawn of growth. The positive control plates (inoculated TSA with 500 ppm *trans*‐cinnamaldehyde) had no apparent bacterial growth. Throughout the experiments, GSE and PSE both easily solubilized in water.

Minimum inhibitory concentration values are presented in Table 2. There were no significant differences (*p* = .26) in the inhibition of PSE (MIC = 51.1 ppm) and GSE (MIC = 47.4 ppm) on the growth of *E. coli* O157:H7. Significantly greater inhibition of *L. monocytogenes* (*p* = .0005) and *S*. Typhimurium (*p* = .01) with GSE than PSE was observed. The average pH of GSE‐containing solutions was 6.6 and the pH of PSE was 6.5 for all concentrations, respectively. Since more inhibition of growth was observed with GSE, all further experiments were completed with GSE.

**Table 2:** Minimum inhibitory concentrations values (ppm) for grape seed extract (GSE) and peanut skin extract (PSE) on growth of *Listeria monocytogenes*,* Escherichia coli* O157:H7, and *Salmonella* Typhimurium at 35°C in vitro

	*L. monocytogenes*	*E. coli* O157:H7	*S. *Typhimurium
GSE	60.6 ± 0.20^a^	47.4 ± 0.40^a^	45.7 ± 0.25^a^
PSE	>68.2 ± ND^b^ *	51.1 ± 0.35^a^	60.6 ± 0.35^b^

Values reported as mean ± *SE*. There was no inhibition of *L. monocytogenes* at any PSE concentration tested.

Different superscript letters within a column indicate significant difference.

*68.2 was the highest concentration tested and there was no inhibition. *SE* was not calculated, therefore is listed; ND, no data.

### 3.4 Compound profile of grape seed extract fractions

The UPLC compound profiles of the GSE found high amounts of catechin (0.06811 mg/mg fraction), epicatechin (0.06265 mg/mg fraction), and epicatechin gallate (0.01870 mg/mg fraction) present in the monomer fraction. The GSE oligomer fraction contained high amounts of dimer B1 (0.02260 mg/mg fraction), dimer B2 (0.01530 mg/mg fraction), and dimer B2 gallate (0.01332 mg/mg fraction). Complete profile of each fraction is presented in Table 3. The monomer and oligomer fractions were highly enriched for monomeric catechins and procyanidins, respectively (Table 3).

**Table 3:** Polyphenol profile for the monomer and oligomer fractions of grape seed extract

Compound	Concentration (μg/mg)		Enrichment factor^a^
	Monomer fraction	Oligomer fraction	
(+)‐Catechin	68.11	2.42	28.1
(−)‐Epicatechin	62.65	3.81	16.4
(−)‐Epicatechin gallate	18.70	0.28	66.8
(−)‐Epigallocatechin gallate	0.20	0.02	10.0
PC dimer B_1_	0.75	22.60	0.0332
PC dimer B_2_	0.79	15.30	0.0516
PC dimer B_5_	1.92	6.77	0.284
PC dimer B_2_ gallate	1.62	13.32	0.122
Unknown PC dimer 1^b^	0.36	6.38	0.0564
Unknown PC dimer 2^b^	0.32	4.54	0.0705
Unknown PC dimer 3^b^	1.62	2.23	0.726
PC tetramer A_2_	6.97	11.46	0.608
PC trimer C_1_	ND	2.55	‐
PC trimer T_2_	4.16	0.13	32.0
PC pentamers	ND	0.39	‐
PC hexamers	ND	2.69	‐

ND, not detected.

a Ratio of the concentration in the monomer fraction compared to the oligomer fraction.

b Likely PC dimers B_3_, B_4_, and either B_6_, B_7_, or B_8._

### 3.5 Impact of monomeric and oligomeric procyanidins on bacterial growth

**Figure 1:**
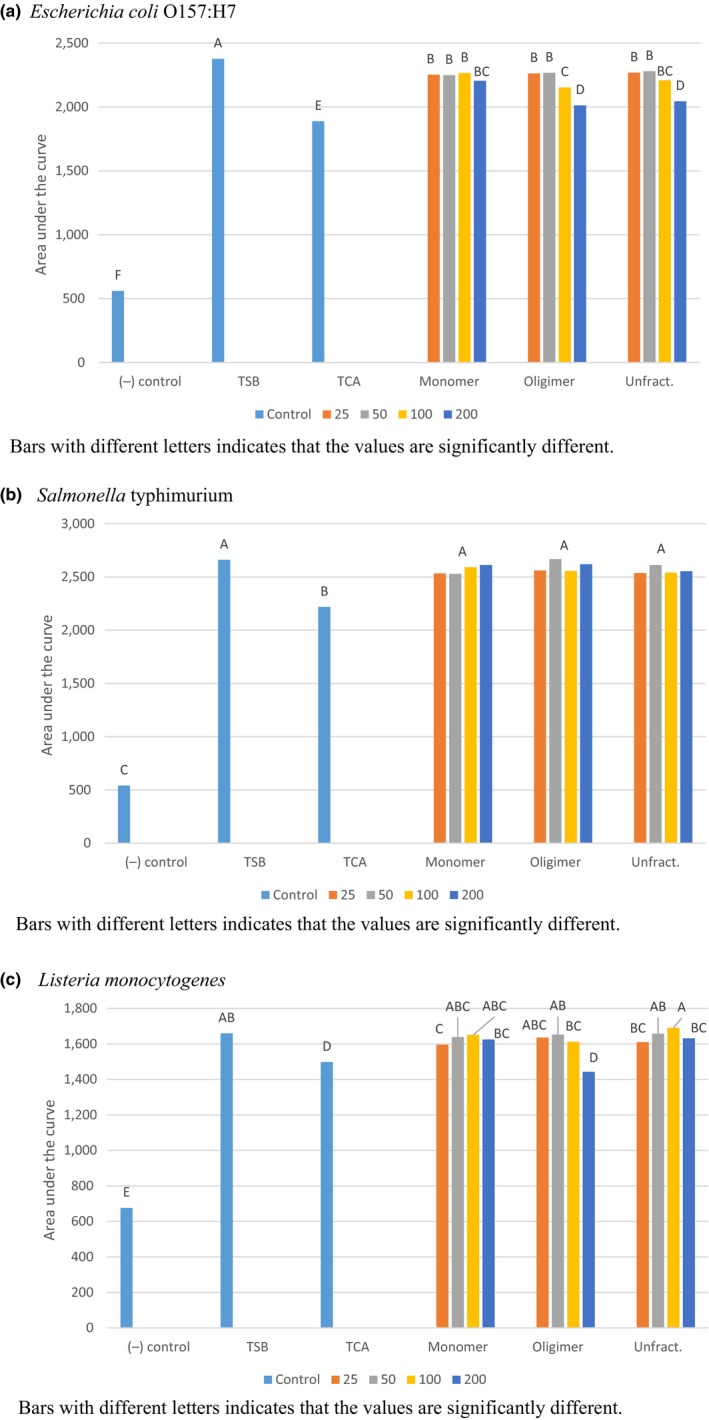
Growth of foodborne pathogens (*Escherichia coli* O157:H7, *Salmonella* Typhimurium, and *Listeria monocytogenes*) alone and coinoculated in tryptic soy broth (TSB) with different concentrations (25, 50, 100, and 200 ppm) of grape seed extract (monomers, oligomers, and unfractionated). Controls include: (−) control, uninoculated TSB; (+) control, inoculated TSB; and *trans*‐cinnamaldehyde (TCA)

The BioScreen provided an optical density reading to assess bacterial growth every 15 min for 48 hr. The AUC were calculated for each curve produced from each fraction/compound combination. The smaller the area, the more inhibition from the compound. The AUC for every organism grown in TSB was significantly greater than the growth in wells with *trans*‐cinnamaldehyde (TCA) and uninoculated TSB (Figure 1a–c). The AUC for every organism grown in TSB was also significantly greater than GSE treatments in all cases except for *S. *Typhimurium (Figure 1b), where there was no effect seen for any treatment except the TCA. In all cases, the AUC for the uninocluated TSB was significantly lower than all other treatments (Figure 1a–c; *p* < .001). All concentrations of GSE (fractionated and unfractionated) significantly reduced the growth of *E. coli* O157:H7, however, higher concentrations performed better (Figure 1a). None of the treatments were as effective as TCA in reducing growth except *L. monocytogenes* was reduced with the oligomeric fraction at the highest concentration (200 ppm), as effective as with TCA (Figure 1c).

## 4 DISCUSSION

### 4.1 Effect of GSE and PSE on *L. monocytogenes*,* E. coli* O157:H7, and *S. *Typhimurium

In our preliminary comparison between GSE and PSE, it was clear that GSE was more successful in inhibiting growth of *L. monocytogenes* and *S. *Typhimurium. It is well documented that GSE possesses antimicrobial properties (Ahn, Grün, & Mustapha, 2004, 2007; Jayaprakasha et al., 2003; Sivarooban et al., 2007; Theivendran et al., 2006), and less documented that PSE possesses these properties (Sarnoski et al., 2012; Yu et al., 2010). This was not surprising, but we wanted to evaluate both extracts using a simple assay to compare the inhibition of low concentrations of both extracts, before determining which extract to fractionate and evaluate further.

Theivendran et al. (2006) reported that high concentrations of *L. monocytogenes* were undetectable after 6 hr of incubation exposed to 1% GSE. Due to the significant drop in populations, this study selected lower concentrations to evaluate. These same researchers also found that the reduction of *L. monocytogenes* in a growth medium significantly increased with increasing concentrations of GSE after 24 hr of incubation at 37°C. These reductions were even greater when nisin was used in addition to GSE (Sivarooban et al., 2007). The biggest differences in MIC values were found to be the Gram‐positive bacteria, which suggests they are more susceptible to the treatments.

Peanut skin extract exhibited no inhibition against *L. monocytogenes* at the highest concentration of >68.2 ppm. To date, no other studies have looked at the effects of PSE on *L. monocytogenes*. PSE has been reported to be active against *E. coli* O157:H7 and *S*. Typhimurium, resulting in complete inhibition, at a concentration of 0.3% w/w (or 3,000 ppm), which is a concentration much higher than the concentration evaluated in this study (Yu et al., 2010). A much lower MIC for *E. coli* O157:H7 and *S*. Typhimurium were found in this study (51.1 and 60.6 ppm, respectively). As previously mentioned, the use of the microplate assay method may be the reason for the differences in the MIC values since Yu et al. (2010) looked at much higher levels 1,000 ppm, 2000 ppm, 3,000 ppm, and 4,000 ppm. Further studies must be performed to investigate the effect of PSE to reduce growth of foodborne pathogens.

For each pathogen, GSE (catechin monomers and B‐type procyanidins) performed better than PSE (catechin monomers and A‐type procyanidins). B‐type procyanidins only contain one single bond between each monomer residue, whereas A‐type procyanidins have two single bonds between each monomer residue. The availability of the chains in the type B procyanidin may allow for more hydroxyl groups that may interfere with the cellular membrane and metabolic processes. The openness may also allow for less intercellular charges that may lead to more interactions with the environment.

Performing the exact same methodologies simultaneously leads to very little variation in conditions and an accurate comparison of the two extracts. However, it is important to note that the present study employed extracts to inhibit growth in liquid media or pour plates. This study did not examine the formation/adhesion of bacterial biofilms, for which A‐type procyanidins have demonstrated superior activity. A possible scenario where an extract high in type A procyanidins may perform better than an extract high in type B procyanidins would be on biofilms on food contact surfaces within a processing plant. In another example, consumption of cranberries (high in type A procyanidins) is recommended to help prevent urinary tract infections since they prevent the attachment of the bacteria to the lining of the urinary tract (Nicolosi, Temperam, Genovese, & Furneri, 2014). Cranberries have a similar concentration of type A procyanidin as peanut skins (Ye et al., 2016).

### 4.2 Effect of fractionated GSE on foodborne pathogens

One study reported GSE MIC values for *E. coli* O157:H7 to be approximately 1,000 ppm, which in this study found to be lower (47.4 ppm) (Jayaprakasha et al., 2003). The lower MIC reported in this study may be explained due to discrepancies in methodology. In our study we used a commercially available extract purchased from Vitaflavan^®^. Jayaprakasha et al. (2003) created their own GSE from raw grape seeds purchased from local processors. Ahn et al. (2004) found that <200 ppm (<0.2 mg/ml) and 4,000 ppm (4.0 mg/ml) prevented the growth of *E. coli* O157:H7 in TSA inoculated at 3.25 and 4.43 log CFU/plate, respectively. Again, the reported MIC is greater than our study, but a different form of GSE was used (Ahn et al., 2004).

### 4.3 Composition of GSE fractions

The UPLC results indicate that the monomer‐rich fraction consisted of catechin, epicatechin, and epicatechin gallate. There were negligible amounts of oligomer compounds in the monomer‐rich fraction. The oligomer‐rich fraction consisted of mostly dimer B1, dimer B2, and dimer B2 gallate, as well as trace amounts of catechin, epicatechin, and epicatechin gallate. These results confirm the technique to separate the GSE monomers and GSE oligomers was successful. The profile of GSE provided by Les Dérivés Résiniques & Terepéniques indicates that it contains approximately 25% monomers and approximately 45% dimers and trimers for a total of approximately 70% of lower degrees of polymerization (Terepéniques, 2009). Since GSE has a negligible amount of polymers, the extract for this study was only fractionated into monomers and oligomers. The UPLC results were consistent with the information provided by Les Dérivés Résiniques & Terpéniques.

Oligomer fractions appeared to inhibit pathogenic growth better than monomer fractions for the three pathogens investigated. This may be because there are more branches for one compound to disrupt the bacterial cell membrane.

The size and number of branch derivatives that oligomers possess may be the biggest factor contributing to the capabilities of GSE as an antimicrobial. Osorio et al. (2010) found the monomer and oligomer fractions from pecan nut shells, pomegranate husks, and creosote bush leaves to be very effective against plant pathogenic fungi. Although this does directly correlate with the research conducted in this study, it shows that individual fractions can thwart growth of pathogens.

It is important to note that the present study examined inhibition of growth only. Procyanidins may be of value for bacteria food systems by a variety of mechanisms, including inhibition of bacterial adhesion to food or equipment surfaces and inhibition of biofilm formation (Daglia et al., 2010; Eydelnant & Tufenkji, 2008; Nicolosi et al., 2014; Percival, Devine, Duggal, Chartron, & Marsh, 2006; Pinzón‐Arango, Holguin, & Camesano, 2011). While our results suggest that GSE was more effective than PSE, the reverse may be true depending on the mechanism studied, as the A‐type procyanidins have demonstrated superior activity compared to B‐type procyanidins for inhibition of bacterial adhesion and biofilm formation (Howell et al., 2005).

It is also necessary to note the differences in total phenolics content between the two extracts employed in the initial antimicrobial screening assay (96.8 ± 1.04 vs. 76.6 ± 7.83% w/w Gallic Acid Equivalents (GAE) for GSE and PSE, respectively). The assays were performed based on the concentration of the extract, rather than the concentration of phenolics as measured by the Folin assay (i.e., ppm extract as opposed to ppm GAE). Therefore, the observed MIC differences between GSE and PSE may be partly due to differences in total concentration of active components. However, a confounding factor is that the measured differences in % total phenolics may be partly attributed to the structural differences between the compounds in the GSE vs. PSE (B‐type vs. A‐type procyanidins), which may affect the Folin assay (Kraus, Yu, Preston, Dahlgren, & Zasoski, 2003; Norris, Preston, Hogg, & Titus, 2011). Additionally, the Folin assay is more accurately a measure of total antioxidant activity as opposed to strictly measuring total phenolics (Everette et al., 2010), and therefore reported differences in total phenolics measured by Folin may be somewhat misleading in situations, such as the present study, where the study outcomes are not related to antioxidant activity.

In conclusion, the relatively low MIC observed in this study suggest that GSE and PSE may be practical, natural antimicrobials for control of foodborne pathogens.
